# Characterization of the complete chloroplast genome of *Centranthera grandiflora* Benth (Orobanchaceae), an important species of medicinal herb

**DOI:** 10.1080/23802359.2021.1934139

**Published:** 2021-05-27

**Authors:** Lan-Ping Zheng, Li-Juan Li

**Affiliations:** a School of Chinese Materia Medica and Yunnan Key Laboratory of Southern Medicinal Resource, Yunnan University of Chinese Medicine, Kunming, China; b Pharmacy Department, Honghe Health Vocational College, Mengzi, China

**Keywords:** Illumina sequencing, phylogenetic analysis, Orobanchaceae

## Abstract

*Centranthera grandiflora* is an important medicinal herb within Orobanchaceae. To date, however, genetic studies on this species remain poor. Here, we assembled the complete chloroplast genome of *C. grandiflora*. Results showed that the genome was 147 655 bp in length, consisting of large and small single copy regions of length 83 550 and 14 891 bp, respectively, separated by two inverted repeat regions of 24 607 bp. Furthermore, the genome contained 132 genes, including 84 protein-coding genes, 39 tRNA genes, and eight rRNA genes. Phylogenetic analysis showed that *C. grandiflora* is closely related to the species of Orobanchaceae. The complete chloroplast genome of *C. grandiflora* should help in the conservation of genetic resources and appropriate utilization of this medicinal herb in the future.

*Centranthera grandiflora* Benth is a medicinal herb and is administered to promote blood circulation and reduce blood stasis (Zhu 2012). However, only few studies have isolated and identified chemical compounds from this species (Liao et al. 2012; Hu 2016) and limited molecular studies have only been carried out recently (Zhang et al. 2019). Therefore, to facilitate genetic studies in the future, we assembled the complete chloroplast genome of *C. grandiflora* in this study.

Fresh leaves were collected from Pingbian, Yunnan, China (103°39′17″E, 23°03′16″N). The specimen was stored at Yunnan University of Chinese Medicine (specimen code YUCM2019044). Total genomic DNA was extracted using a Tiangen Plant Kit (Beijing, China). A purified genomic DNA library was constructed and sequenced using the Illumina NovaSeq 6000 Platform (Benagen Tech Solution Co., Ltd, Wuhan, China). Genome was assembled using SPAdes v3.6.1 (Bankevich et al. 2012). The annotation was performed with Plastid Genome Annotator (PGA) (Qu et al. 2019).

The complete genome of *C. grandiflora* was 147 655 bp in length, consisting of large and small single copy regions of length 83 550 and 14 891 bp, respectively, separated by two inverted repeat regions of 24 607 bp. The complete chloroplast genome of *C. grandiflora* displayed the typical quadripartite structure of most angiosperm chloroplast genomes. The overall GC content was 38.24%. Furthermore, the genome contained 132 genes, including 84 protein-coding genes, 39 tRNA genes, and eight rRNA genes.

Phylogenetic analysis was performed based on the complete chloroplast genome of *C. grandiflora* and 25 related species (Figure 1). MAFFT v7 was used to align the sequences (Katoh and Standley 2013), and RAxML v8.2.10 was used to construct a maximum-likelihood (ML) tree (Stamatakis 2014). Results showed that *C. grandiflora* formed a clade with species within Orobanchaceae with 100% support (Figure 1). *Centranthera grandiflora* was originally classified in Scrophulariaceae in the Flora of China (Editorial Committee of Flora of Chinese Academy of Sciences 1990). With the split of the Scrophulariaceae following the molecular analyses, *Centranthera* was classified into the Orobanchaceae (Chen et al. 2016). Our phylogenetic analysis confirms this placement. The complete chloroplast genome of *C. grandiflora* should help in the conservation of genetic resources and appropriate utilization of this medicinal herb in the future.

**Figure 1.**
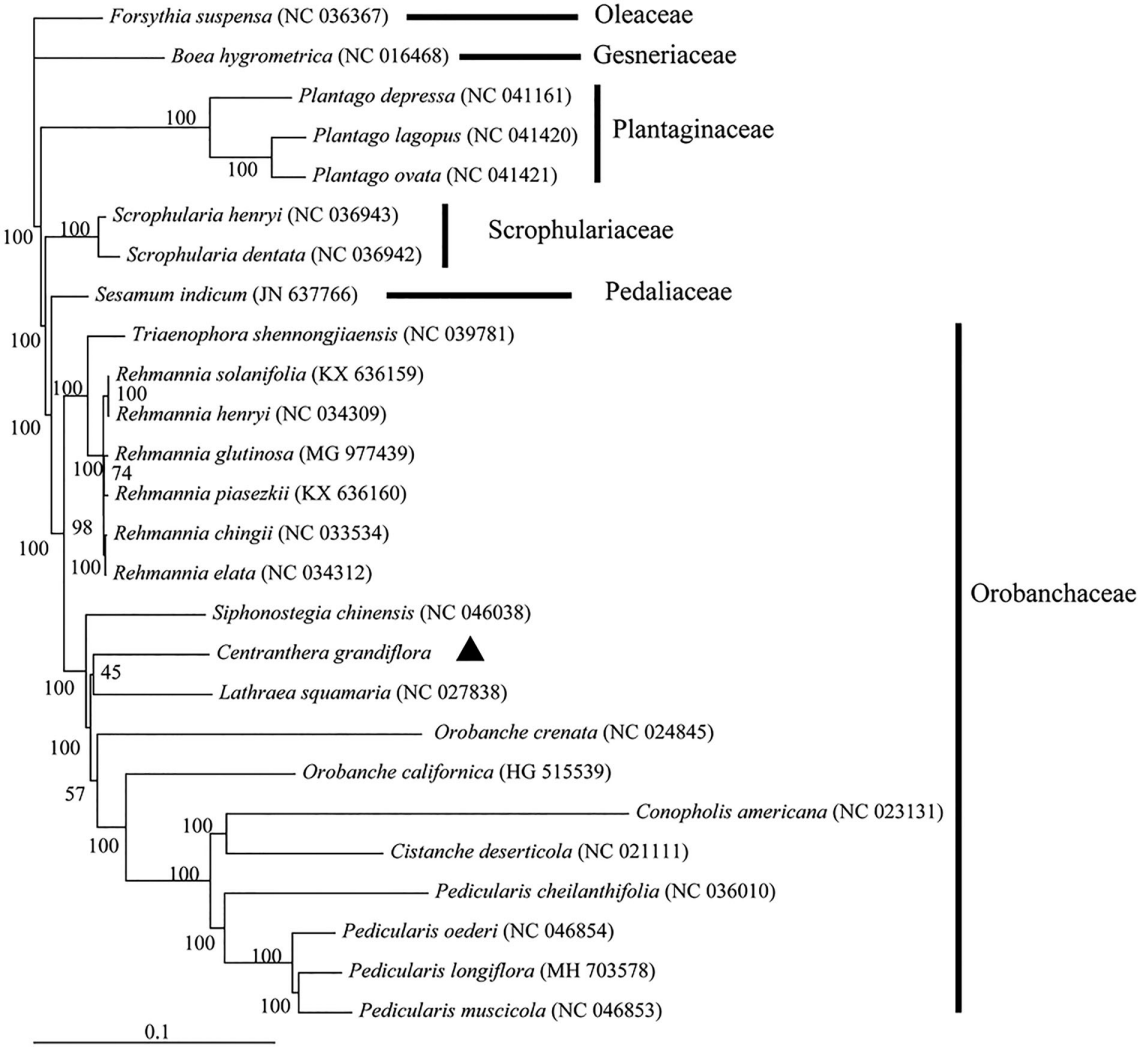
Phylogenetic tree of *Centranthera grandiflora* and 25 related species based on complete chloroplast genomes. Nodal numbers are ML bootstrap values.

## Data Availability

The genome sequence data of this study are openly available in GenBank of NCBI at (https://www.ncbi.nlm.nih.gov/) under the accession no. MW262988. The associated BioProject number is PRJNA706067.
